# The Added Value of [^18^F]Choline PET/CT in Low-Risk Prostate Cancer Staging: A Case Report

**DOI:** 10.3390/life12111728

**Published:** 2022-10-28

**Authors:** Antonio Piras, Riccardo Laudicella, Luca Boldrini, Andrea D’Aviero, Antonella Sanfratello, Antonino La Rocca, Salvatore Scurria, Giuseppe Salamone, Pierpaolo Alongi, Tommaso Angileri, Antonino Daidone

**Affiliations:** 1 UO Radioterapia Oncologica, Villa Santa Teresa, 90011 Bagheria, Italy; 2 UO Medicina Nucleare, Fondazione Istituto G. Giglio, 90015 Cefalù, Italy; 3 Nuclear Medicine Unit, Department of Biomedical and Dental Sciences and Morpho-Functional Imaging, University of Messina, 98121 Messina, Italy; 4 UOC Radioterapia Oncologica—Fondazione Policlinico Universitario Agostino Gemelli IRCCS, Dipartimento di Diagnostica per Immagini, Radioterapia Oncologica ed Ematologia, 00168 Roma, Italy; 5 Radiation Oncology, Mater Olbia Hospital, 07026 Olbia, Italy; 6 Università degli Studi di Palermo, Radioterapia Oncologica, 90133 Palermo, Italy; 7 UOC Urologia, P.O. Paolo Borsellino, 91025 Marsala, Italy; 8 UOC Urologia, Azienda di Rilievo Nazionale ad Alta Specializzazione “Civico Di Cristina Benfratelli”, 90127 Palermo, Italy; 9 UOC Chirurgia Urologica, Fondazione Istituto G. Giglio, 90015 Cefalù, Italy; 10 UO Radiologia, Villa Santa Teresa, 90011 Bagheria, Italy

**Keywords:** prostate cancer, radiotherapy, [^18^F]Choline PET/CT

## Abstract

In the management of prostate cancer (PCa), correct staging is crucial in order to assess the right therapeutic approach. [^18^F]Choline PET/CT has been shown to provide more accurate staging information than conventional imaging approaches. The aim of this paper is to provide a real practice demonstration of the impact of [^18^F]Choline PET/CT on low-risk prostate cancer staging and clinical management. We report a 64-year-old man with biochemical PCa recurrence diagnosis after transurethral resection of the prostate. The patient, after the detection of an increased level of PSA, underwent multi-parametric prostate magnetic resonance imaging (mpMRI) that did not show evidence of disease. The patient was admitted to perform [^18^F]Choline PET/CT that showed a macroscopic prostate recurrence. Patient underwent photon external beam radiation therapy (EBRT) treatment, and [^18^F]Choline PET/CT was also used to define treatment volumes. At 3- and 6-month clinical follow-up evaluations, no late toxicity was detected and a significant reduction in PSA value was shown. Therefore, our case highlights the potential usefulness of [^18^F]Choline PET/CT for the staging of low-risk prostate cancer and its impact on the management and quality of life of such patients. The presented case should urge the scientific community to enhance larger and multicentric studies, assessing more extensively the potential impact of [^18^F]Choline PET/CT in this clinical scenario.

## 1. Introduction

Prostate cancer (PCa) is one of the primary causes of cancer diagnosis worldwide [1]. Correct staging with an accurate definition of disease extension is crucial to assess prognosis and better optimize therapeutic strategies. In this scenario, several studies have shown that clinical staging could underestimate PCa when compared with post-surgical staging [2,3].

Conventional PCa staging includes rectal examination, prostate biopsy, contrast-enhanced computed tomography (CT) of the chest–abdomen, multi-parametric prostate resonance imaging (mpMRI) and a [^99m^Tc] bone scan, according to the risk class [4,5,6,7].

More recently, new imaging modalities have been implemented in order to improve the overall accuracy of staging, such as positron emission tomography/CT (PET-CT) with different [^18^F] radionuclides, namely, choline and prostate-specific membrane antigens (PSMAs), resulting in superior outcomes to conventional image-based staging [8].

Namely, in a head-to-head comparison for PSA values above 2.0 ng/mL, the detection rate resulted in 85% for [^68^Ga]Ga-PSMA PET/CT versus 60% for [^18^F]Choline which, however, is still the more widely available option in Italy [9].

Nonetheless, the limited scientific evidence does not, to date, support the systematic use of PET-CT for PCa staging [7,10,11].

This case report aims to emphasize the added value of [^18^F]Choline PET-CT in low-risk PCa staging.

## 2. Case Report

A 64-year-old Caucasian man in good general clinical condition was referred to our radiation therapy (RT) department for a high-risk biochemical recurrence (BCR) according to EAU guideline classifications with a PSA level increase up to 6.96 ng/mL after an incidental PCa diagnosis following transurethral resection of the prostate (TURP) [12]. Seven months earlier he had undergone TURP for benign prostatic hyperplasia with a PSA value of 6.2 ng/mL. The histological examination documented a stage pT1a low-grade prostate adenocarcinoma ISUP 1 (Gleason score 6, 3+3). After TURP, the PSA value was 1.9 ng/mL.

**Figure 1:**
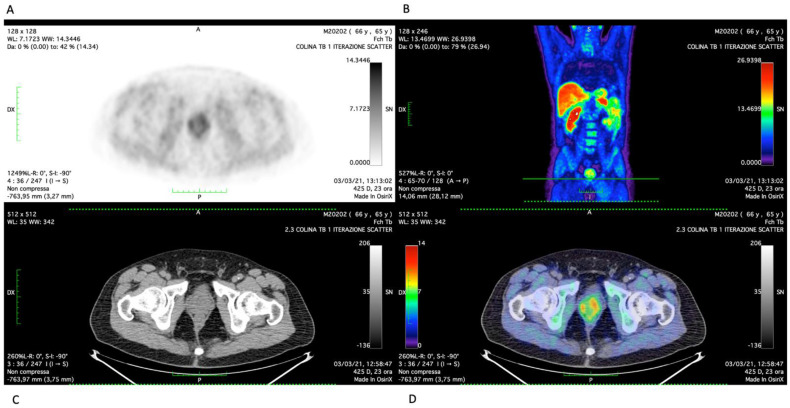
[^18^F]Choline positron emission PET-CT scan; (**A**) attenuation-corrected (AC) view; (**B**) maximum-intensity projection (MIP) view; (**C**) axial CT view; (**D**) axial fused view.

The patient then underwent staging examinations with a mpMRI of the prostate which did not show evidence of disease. Following EAU guideline indications, a [^18^F]Choline positron emission PET-CT was performed, revealing suspicious intraprostatic uptake (Figure 1).

Following multidisciplinary discussion with the referring urologist, it was decided to perform a new biopsy, but the patient refused to undergo the procedure. The clinical approach was further discussed by a multidisciplinary tumor board, and a radical RT approach was proposed to the patient, who was informed of the risks of this approach. No androgen deprivation therapy was started.

The patient, therefore, underwent photon external beam RT (EBRT) to the prostate and seminal vesicles.

The simulation CT (slice thickness 2.5 mm) was performed with a supine position set-up, with the arms crossed over the chest, and the legs immobilized on an appropriate pelvic repositioning system (Combifix™). The patient’s preparation protocol required drinking 500 mL of water 30 min before the CT and performing a rectal enema. The simulation CT images were then co-registered with the mpMRI and PET images, in order to accurately identify the target volumes and generate the treatment plan.

The patient was treated on a Elekta Synergy^®^ linear accelerator equipped with an 80-leaf multilamellar collimator and an integrated Cone Beam (kV CBCT) system. The treatment plan was calculated using Pinnacle3 software vers. 16.02 (Philips, The Netherlands) and the preferred planning technique was volumetric-modulated arc therapy (VMAT) with simultaneous integrated boost (SIB). The prescribed dose was 70 Gy on the prostate in 28 sessions of 2.5 Gy, and 57.4 Gy on the seminal vesicles in 28 sessions of 2.05 Gy; there were five fractions per week. The clinical target volume (CTV) was contoured according to the “Carcinoma of the Prostate Guidelines—AIRO, 2016”; the planning target volume (PTV) was obtained by adding 8 mm to the CTV in all directions except posteriorly, where it was of 6 mm [13].

The 95% coverage of the prescribed dose was 98.9% on the CTV and 97.8% on the PTV.

During RT, the patient underwent weekly clinical examinations for early toxicity onset monitoring. Ten days after starting RT, the patient decided to discontinue treatment due to the appearance of grade 2 rectal tenesmus according to the Common Terminology Criteria for Adverse Events (CTCAE) v5.0 scale [14]. After 3 days of corticosteroid therapy, the patient resumed the treatment which was completed without further interruptions. During treatment, grade 1 pollakiuria appeared about halfway through the sessions.

At the three- and six-month follow-up examinations, the patient did not show late toxicity, and the PSA values were 2.04 and 1.86 ng/mL, respectively.

## 3. Discussion

This case report demonstrates that the use of [^18^F]Choline PET-CT staging had a dramatic impact on the management of a PCa patient. In this scenario, the use of [^18^F]Choline PET-CT offered a safer treatment option to the patient. The use of PET-CT instead of conventional imaging (CT + bone scintigraphy) may avoid the need for the patient to undergo two procedures (bone scintigraphy and CT), also reducing waiting time, social costs and radiation exposure [15,16]. Furthermore, with regard to RT, PET-CT appeared to be particularly useful for RT contouring of the prostate and the confirmation of treatment volumes [17,18,19]. In this context, the use of simulation PET-CT to improve target contouring is becoming popular [20,21]. In this framework, biology-guided radiotherapy (BgRT) has recently been introduced as a new external beam radiotherapy technique that combines PET-CT with a 6 MV linear accelerator, paving the way to a brand new interpretation of hybrid technologies in RT [22]. Results from the FLAME Randomized Phase III Trial demonstrated that a high focal boost strategy to improve tumor control while respecting the organ at risk with dose constraints is effective and safe [23]. PET-CT allows the identification of the area to be boosted and we expect its use to increase progressively in clinical practice as a reliable support for target segmentation and planning. The major limitation of PET–choline is the existence of a significant overlap between PCa and benign prostatic hyperplasia [24]. A very recent study demonstrated that ^11^C-choline PET/CT-based multi-metabolic parameter combination can help break this limitation [25]. In comparison with choline, PSMA represents an “ideal” biomarker because it is markedly overexpressed by most PCa cells, with a low presence in the bloodstream (transmembrane localization); furthermore, PSMA expression showed a positive correlation with PCa grading and aggressiveness [26]. Therefore, PSMA PET is characterized by a high sensitivity and specificity with reduced false-positive and false-negative results, improving the confidence also for PCa initial diagnosis and resulting in more reproducible results than mpMRI [27,28].

Despite a superior accuracy for low-risk PCa and its growing acceptance as a staging tool [29], PSMA is still not approved worldwide and some countries are still using choline PET-CT for PCa BCR. The former represents a valid opportunity for low-risk patient candidates for radiotherapy, especially considering the heterogeneity of PCa and new knowledge on imaging correlation with histopathological patterns [22,23,24,25].

Clinicians should be aware of the potential impact of such modality on low-risk prostate cancer staging.

## Data Availability

The data presented in this study are available on request from the corresponding author.
